# The Joint Simon task is not joint for capuchin monkeys

**DOI:** 10.1038/s41598-024-55885-x

**Published:** 2024-03-11

**Authors:** Mayte Martínez, Matthew H. Babb, Friederike Range, Sarah F. Brosnan

**Affiliations:** 1 Domestication Lab, Konrad Lorenz Institute of Ethology, University of Veterinary Medicine Vienna, Savoyenstraße 1a, 1160 Vienna, Austria; 2 Language Research Center, Georgia State University, Atlanta, GA 30034 USA; 3 Departments of Psychology and Philosophy, Neuroscience Institute, Georgia State University, Atlanta, GA 30034 USA

**Keywords:** Psychology, Evolution

## Abstract

Human cooperation can be facilitated by the ability to create a mental representation of one’s own actions, as well as the actions of a partner, known as action co-representation. Even though other species also cooperate extensively, it is still unclear whether they have similar capacities. The Joint Simon task is a two-player task developed to investigate this action co-representation. We tested brown capuchin monkeys (*Sapajus [Cebus] apella*), a highly cooperative species, on a computerized Joint Simon task and found that, in line with previous research, the capuchins' performance was compatible with co-representation. However, a deeper exploration of the monkeys’ responses showed that they, and potentially monkeys in previous studies, did not understand the control conditions, which precludes the interpretation of the results as a social phenomenon. Indeed, further testing to investigate alternative explanations demonstrated that our results were due to low-level cues, rather than action co-representation. This suggests that the Joint Simon task, at least in its current form, cannot determine whether non-human species co-represent their partner’s role in joint tasks.

## Introduction

The animal kingdom abounds with instances of cooperation between individuals, such as group-hunting, territorial defense, or cooperative breeding^1^. Functionally cooperative outcomes can be achieved by different cognitive processes^2^. In some instances, cooperation may hinge on low-level environmental cues that prompt a similar response from all the individuals (e.g., the movement of a potential prey that prompts all individuals to pursuit^3^). At the other end of the continuum, humans understand their partner’s role and intentions in the task^4^, which enables them to finely tune their behaviour to each other. One proposed mechanism underlying human cooperation is *action co-representation*, which refers to the ability of an individual to create a mental representation of a task that integrates both their own and their partner’s actions^5^. Action co-representation can facilitate cooperation because it allows subjects to predict their partners’ movements and flexibly adjust their own movements accordingly^6^. By studying action co-representation across different species, we can gain insights into the evolutionary origins of humans’ exceptional abilities to cooperate, and identify which mechanisms are unique (or not) to humans.

**Figure 1:**
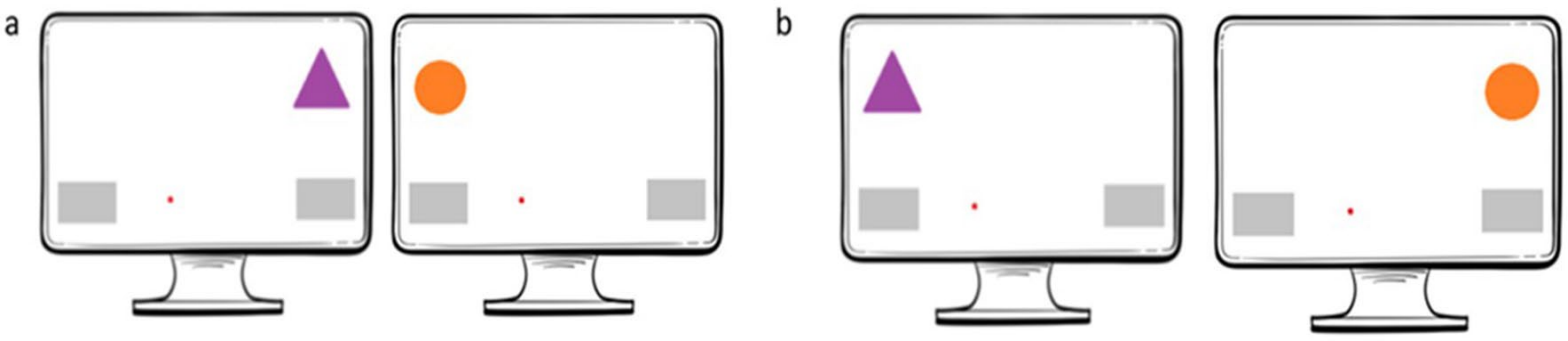
Examples of compatible (**a**) and incompatible trials (**b**). Note that when the orange circle is presented, the correct response is to move the cursor to the left, and when the purple triangle is presented, the correct response is to move the cursor to the right. The red dot represents the cursor on the screen and the gray rectangular shapes are the response boxes.

Action co-representation might enhance coordination in tasks in which both individuals need to perform identical actions to reach a common goal (e.g., two people transporting a heavy box together, holding it and moving in the same direction at the same time). However, when actors are required to perform complementary roles (e.g., each individual is responsible for a different part of the task), action co-representation can hamper joint performance, as the representation of the other individual’s action in the task conflicts with the representation of one’s own actions. The measurement of these conflicts is frequently used to quantify action co-representation, in joint interference tasks such as in the Joint Simon task^7^. The Joint Simon task (explained below) is a social version of the Simon task. In the Simon task, individuals must make responses that may be spatially congruent or incongruent, potentially setting up a conflict between the correct response and the stimuli’s spatial orientation^8^. Specifically, individuals are required to give spatial responses to non-spatial features of certain stimuli, for instance pressing the right key when an orange stimulus is shown on the screen, and the left key for a purple stimulus. The core aspect of the task is that the stimulus target can be shown randomly on the right or on the left side. The location of the stimuli is not relevant for the task, but subjects usually show faster responses and fewer errors when the stimulus is on the same side as the key that needs to be pressed. In the example above, subjects should be faster if the orange stimulus is shown on the right side of a screen (compatible trial) than if it is shown on the left side (incompatible trial) (see Fig. 1).

This effect, known as the “compatibility effect” or “Simon effect”, is explained by the mismatch between the spatial representation of the stimulus location and the response location. That is, when the stimulus target is shown, subjects build a mental representation of both the stimulus and the correct response, both including spatial coordinates (i.e., location of the stimulus and location of the response). If the spatial representations of stimulus and response are different, the brain takes additional time to discriminate which one corresponds to the appropriate answer to be executed (for an overview of various explanations for this phenomenon see^9^). Indeed, the compatibility effect does not appear in the Half task (also known as the Go/No-go task), which is identical to the Simon task except that subjects must only respond to one stimulus and thus only have access to one response box to press when that stimulus is shown (“go trials”). If the other stimulus is shown they are supposed to ignore it (“no-go trials”)^7^. The absence of a compatibility effect in the Half task is explained by the lack of conflict between spatial representations (e.g., subjects choose whether to answer or not, instead of choosing whether the correct answer is right or left).

Finally, the Joint Simon task is a social version of the task in which two subjects perform the complementary Half tasks together, which adds up to the full Simon task but achieved by two partners that are playing jointly. Thus, in this Joint Simon task, individuals are tested in pairs and each participant has access to one of the two response options, so they have to respond to only one stimulus (i.e., participant 1 responds when the orange stimulus is shown, and participant 2 responds when the purple stimulus is shown), while ignoring the other. Because the task requirements of the Joint Simon and the Half task are the same (i.e., one of the answers is not available for the actors in both conditions), one might expect to obtain similar results in both tasks in terms of compatibility effect. Importantly, studies have found that participants do show a compatibility effect in the Joint Simon task similar to the one found in the Simon task^7,10^. That is, subjects are faster/more accurate in selecting a response when the stimulus location is compatible with the correct response. In the Simon task, the compatibility effect is not interpreted as a social phenomenon per se, but rather a result of how the brain processes different aspects of a task^11^. The occurrence of the compatibility effect in both the Joint Simon and the Simon task suggests that participants are processing the same aspects of the task in both conditions. This occurs despite the fact that, in the Joint Simon task, subjects are responsible for only half of the task and they are supposed to disregard the other half. Researchers have proposed that this compatibility effect emerges in the Joint Simon task because subjects have a mental representation of both their role in the task and their partner’s^7,12,13^. Thus, the appropriate responses are not represented anymore as “Response/No response” as in the Half task, but as “If the correct response is the Right, then I should respond/If the correct response is the Left, then my partner should respond”. If participants are indeed considering the actions of their partners (action co-representation), then the correct response is again represented in spatial terms (as in the Simon task), which would explain the compatibility effect.

The Joint Simon task has been extensively used to study co-representation in humans, including adults^7^, children/adolescents^14,15^, and clinical populations^16^. In recent years, three non-human primates have also been tested in the Joint Simon task: marmoset monkeys^17^, capuchin monkeys, and Tonkean macaques^18,19^. All of them showed a compatibility effect in the Joint Simon task, suggesting that co-representation might be shared at least across haplorhine primates^6^. Nevertheless, whereas humans are typically tested in the Joint Simon task using computer-based setups, monkeys in^17,18^ had to interact with mobile platforms that the animals had to approach and pull in response to auditory stimuli. This is very different from the typical human format and raises the question of whether these species would similarly exhibit motor co-representation signs under conditions more akin to the human studies. Indeed, research has shown that capuchin monkeys’ performance and learning speed is affected by the modality (real food and objects vs computer images) in which the task is presented^20^. Moreover, the spatial disposition of the experimental setup has an effect in the compatibility effect^21–23^. These findings prompted some researchers to attribute the compatibility effect in the Joint Simon task to low-level perceptual cues rather than co-representation^24,25^. Hence, a similar computer-based setup is crucial for ensuring comparable interpretations of results between human and non-human.

In this study, we aimed to test whether brown capuchin monkeys (*Sapajus [Cebus] apella*) show the compatibility effect in a computer based Joint Simon task. Capuchin monkeys are an established model species for studies on social cognition^26^ and they understand, at least to some extent, the contingencies of cooperation. For instance, capuchin monkeys are sensitive to the presence of a partner in a cooperative pulling task^27^, and match their partner’s choices in coordination games^28,29^. In addition, this species has previously shown the compatibility effect in a manual Joint Simon task^19^, making them ideal for a closer comparison to human methodology.

**Figure 2:**
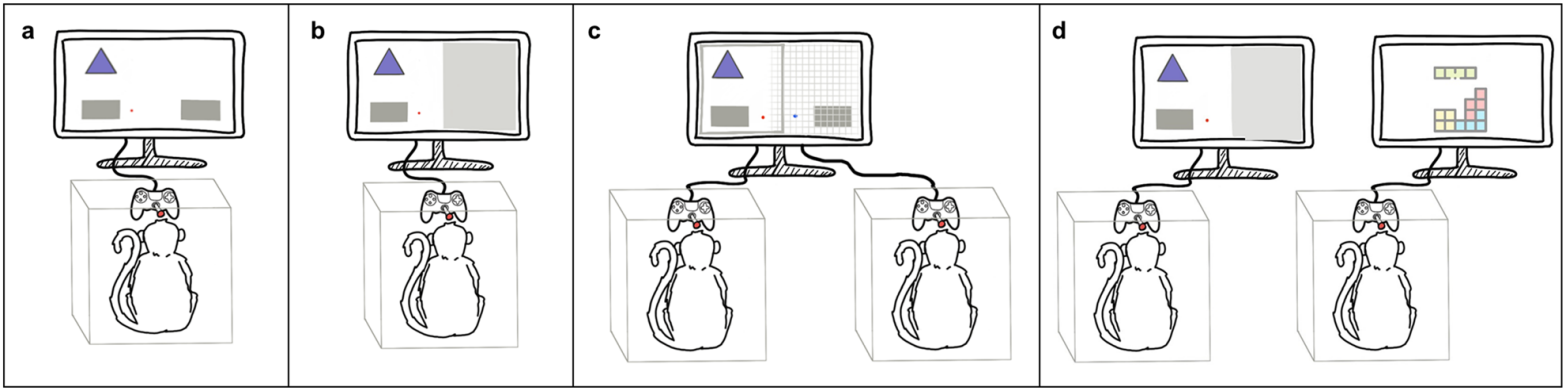
Schematic representation of the setup for the four conditions in Experiment 1. In the Simon task (**a**), subjects solved the task alone (i.e. they could move the cursor to either of the two response boxes in response to the stimuli). In the Half task (**b**), only one response box is available and thus, subjects performed half of the Simon task (i.e. selecting the response box when one stimulus is shown and not giving any response when the other stimulus is shown). If subjects successfully refrained from selecting a response when the correct response box was not available, the answer was coded as correct, but they did not receive a reward. In the Joint Simon task (**c**), subjects were tested in dyads, and each individual was responsible for half of the task (i.e., they could only move the cursor to one of the two response boxes). Subjects were rewarded for both their correct choices and their partner’s correct choices. In the Social facilitation task (**d**), subjects performed the Half task with their partner present but engaged in a different task on a separate computer. Shaded area represents the screen area in which subjects can not move the cursor. In addition, in (**c**) the areas in which first and second subject can move the cursor are delimited by a gray line and gridded area respectively (note that both overlap in the middle of the screen). In Experiment 2, the Half task with unrewarded correct-no answers was identical to the Half task in Experiment 1 (**b**), as was the Half task with rewarded correct answers, except that unlike (**b**), subjects were rewarded for correct no-answers.

We tested 10 socially housed brown capuchin monkeys (8 females, 2 males) in 7 different dyads, using a computer-based setting in which they could select a response with a joystick. In Experiment 1, we tested whether the monkeys showed a compatibility effect in four different conditions: the Simon and the Joint Simon task, and, as control conditions, the Half and Social facilitation task (see Fig. 2). If capuchin monkeys co-represent their partners actions, we should find the compatibility effect in the Simon and Joint Simon tasks, but not in the Half and Social facilitation tasks.

After completing Experiment 1, we discovered that in the Half (and Social facilitation) task, the capuchins selected the available box in virtually every trial, independently of the stimulus shown, which is in line with results from the other non-human primates tested in this paradigm^17,18^ and consistent with studies showing that non-human primates have difficulties suppressing prepotent responses (e.g.,^30,31^). Nevertheless, it is difficult to disentangle whether non-human primates were unable to refrain from selecting a response, or whether they merely did not understand the task as a Go/No-go condition and/or were not paying attention to the stimuli shown. This last explanation is especially plausible considering that primates in the Language Research Center (and likely in other facilities) participate in a variety of tasks on a daily basis. Hence, without explicit training it might be difficult for them to understand that the Half task is a Go/No-go version of the Simon task, and thus, they behave as if they were playing a completely new task. We addressed this issue in Experiment 2, in which we first trained subjects on a Go/No-go (GNG) task to ensure that they understood the Half tasks. For the GNG task, subjects had to respond to one type of stimulus (“go trials”) while refraining from responding to the other type (“no-go trials”). Afterwards, we tested them in two versions of the Half task: one identical to the Half task in Experiment 1, and a second version in which subjects received rewards for correctly inhibiting a response, to control for the influence of the reward pattern (in the Joint Simon task, subjects are rewarded for both their own and their partners’ correct responses). We expected that after the training, in Experiment 2, subjects would understand that they are required to respond to only half of the trials in the Half task and treat it as a ‘real’ GNG task. This, together with the modification of the reward pattern, would result in a Half condition in which the subject would need to do the same as in the Joint Simon, just without a partner, making it a better control. If subjects still did not show a compatibility effect in Experiment 2, it would support the interpretation of action co-representation in the Joint Simon task in Experiment 1.

## Results

### Experiment 1: is there a compatibility effect across conditions?

We tested whether there was a compatibility effect in the different conditions using a General Linear Mixed Model (GLMM) with binomial error distribution and monkey ID as a random intercept. Our response variable was whether the subject’s choice was correct in each trial. As in^17,18^, in the case of the Half and Social facilitation task we also considered trials to be correct if subjects correctly refrained from giving a response when the correct response box was not available. Because we expected a compatibility effect only in the Simon and Joint Simon condition, we included the interaction between compatibility (compatible vs incompatible trials) and condition (Simon, Joint-Simon, Half, and Social facilitation) as a predictor. To account for possible learning effects, we included two further fixed factors: trial number and session number (see Methods).

**Figure 3:**
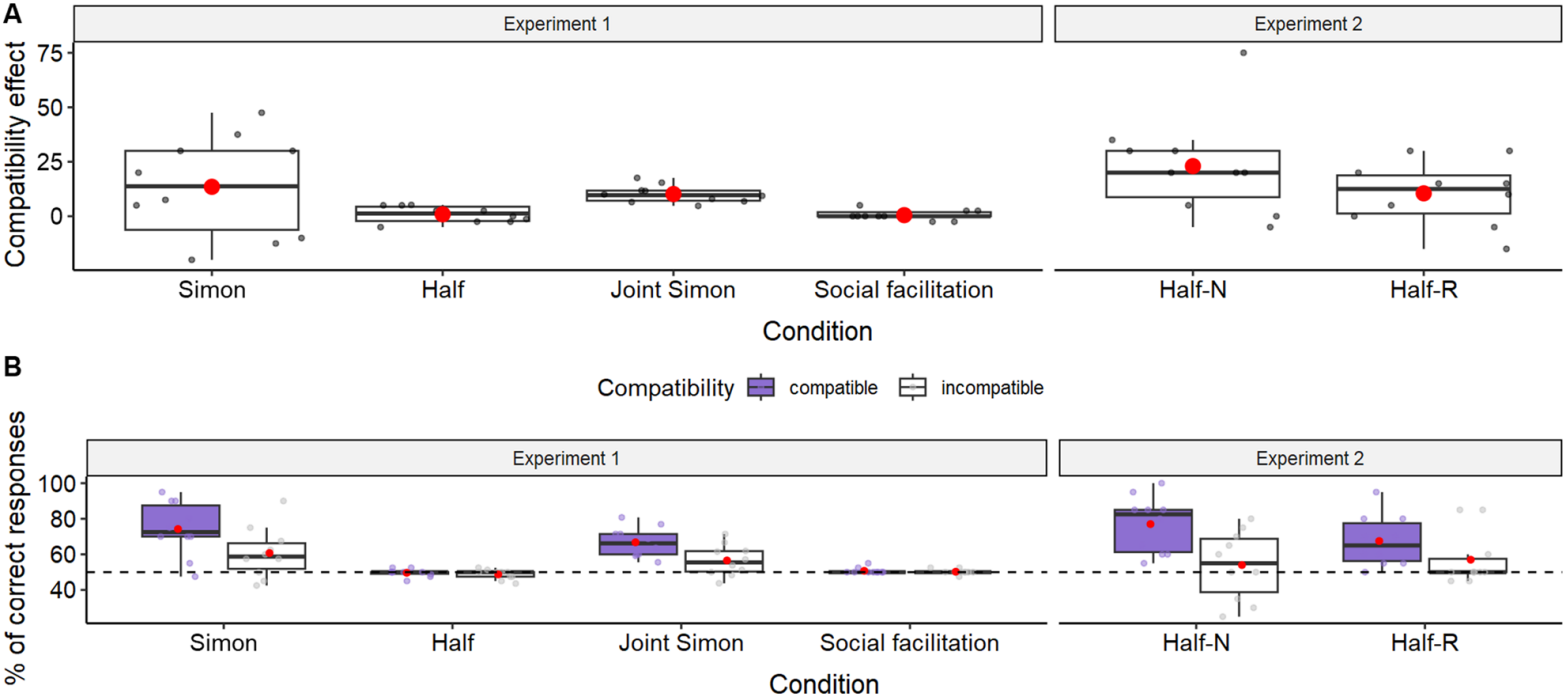
Results of Experiment 1 and Experiment 2. (**a**) Compatibility effect (% of incorrect choices in incompatible trials minus compatible trials) and (**b**) Percentage of correct responses, in all the conditions of Experiment 1 and Experiment 2. Boxes and whiskers show the median value and the lower and upper quartile scores. Mean values are represented by a red circle. The dots display the average by subject. In (**b**) the dashed line shows the chance level (50%). *Half-N* half task with non-rewarded correct no-responses, *Half-R* half task with rewarded correct no-responses.

The comparison between the full and the null model (identical to the full model but lacking the predictor: interaction term between compatibility and condition), revealed that the interaction between compatibility and condition had a significant effect on the proportion of correct trials (*χ2* = 13.666, *df* = 3, *p* = 0.003, see Table 1 and Fig. 3), meaning that there was a compatibility effect, but that it depended on the condition. Specifically, comparison of the estimated marginal means for compatible and incompatible trials across the four conditions revealed that there was a compatibility effect in the Simon (estimate ± se = 0.699 ± 0.163, p < 0.001) and Joint-Simon tasks (estimate ± se = 0.382 ± 0.187, p = 0.041), but not in the Half (estimate ± se = 0.025 ± 0.152, p = 0.869) or the Social facilitation tasks (estimate ± se = 0.020 ± 0.152, p < 0.894), as predicted.

**Table 1:** Experiment 1: GLMM of proportion of correct trials predicted by condition and compatibility.

	Estimate	SE	CI lower	CI upper	Model stability min	Model stability max	*χ2*	*df*	*p*^1,2^
Intercept	1.073	0.121	0.851	1.354	0.950	1.247			
Condition (half)^3^	− 1.089	0.156	− 1.415	− 0.798	− 1.241	− 0.969			
Condition (joint)^3^	− 0.419	0.182	− 0.770	− 0.058	− 0.581	− 0.223			
Condition (social facilitation)^3^	− 1.041	0.156	− 1.366	− 0.735	− 1.211	− 0.915			
Compatibility (incompatible)	− 0.699	0.163	− 1.024	− 0.387	− 0.866	− 0.522			
Trial number^4^	0.012	0.039	− 0.073	0.093	− 0.005	0.030	0.102	1	0.749
Session number^4^	0.038	0.039	− 0.041	0.114	0.024	0.057	0.957	1	0.328
Condition (half)*compatibility (incompatible)	0.674	0.214	0.266	1.120	0.517	0.824	13.666	3	**0.003**
Condition (joint)*compatibility (incompatible)	0.317	0.240	− 0.189	0.797	0.139	0.493			
Condition (social facilitation)*compatibility (incompatible)	0.679	0.214	0.243	1.106	0.511	0.842			

^1^Statistically significant p-values are shown in bold.

^2^Some p-values are not indicated due to their limited interpretation.

^3^Reference categories: condition = Simon task; compatibility = compatible.

^4^Predictors were z-transformed to a mean zero and a standard deviation of one. Original mean(sd): trial number = 20.518 (11.561); session number = 1.472 (0.499).

Our results correspond with the pattern expected if the capuchin monkeys co-represented each other's actions in the Joint Simon task, that is, we found a difference between compatible and incompatible trials only in the Simon and the Joint Simon tasks and not in the two control tasks. Yet, when we plotted not only this difference (as it is typical in this paradigm^17,18^, see Fig. 3a), but also the absolute proportion of successful trials (see Fig. 3b), we observed a concerning anomaly. While in the Simon and Joint Simon tasks the proportion of success varied across subjects and whether the trial type was compatible or incompatible, in the Half and Social facilitation tasks, subjects maintained a consistent 50% accuracy across all trials, that is, they kept their success exactly at chance level irrespective of trial type. Upon further inspection of the monkeys’ responses in the Half and Social facilitation tasks, we found that they consistently selected the available response box in nearly all the trials, meaning that they failed the trials in which the correct response box was not available (hence the overall chance levels of choice). The percentage of trials in which they selected nothing was consistently low in all conditions: 1.9% in the Simon task, 4.2% in the Joint Simon task, 1.8% in the Half task, and 0.8% in the Social facilitation task, which corresponds to only 0 to 3 trials in each 40-trial session. That is, monkeys almost always chose the available response box regardless of the stimulus target, which implies a lack of understanding of the task and challenges the validity of the Half and Social facilitation tasks as control conditions. As discussed above, we then ran Experiment 2 post hoc to explore this anomaly.

### Experiment 2: Could our results in Experiment 1 be explained by a lack of understanding of the Half task, instead of co-representation?

In this experiment, subjects were trained on a Go/No-go (GNG) task and retested in the Half task, which led to an increase in no-response trials, as designed. No-responses went from virtually none in the Half task in Experiment 1 to more than one quarter of the trials in Experiment 2. Moreover, no-responses in Experiment 2 did not increase arbitrarily, but there were clearly more trials in which the subject selected nothing (no-response) when the correct option was not available than when they could select the correct response box (27% vs 2.5% in the Half task in which correct no-responses were not rewarded; and 37.5% vs 6.5% in the Half task in which correct no-responses were rewarded). This suggests that monkeys were paying attention to the stimulus shown and responding accordingly.

We used the same GLMM as in Experiment 1 to test whether there was a compatibility effect in these two versions of the Half task, including task version (rewarded or unrewarded correct no-responses), trial number and task order as additional fixed effects. When we compared this model with a model lacking the predictors, we found that the likelihood of success was influenced by the trial compatibility (*χ2* = 10.816, *df* = 2, *p* = 0.004, see Table 2 and Fig. 3). Specifically, subjects were more likely to succeed in compatible trials than in incompatible trials (estimate ± se = − 1.116 ± 0.301, *χ2* = 7.19, *df* = 1, *p* = 0.007). Additionally, the compatibility effect seemed to be stronger in the version of the task in which subjects were not rewarded for correct no-responses (see Fig. 3). Nevertheless, neither the interaction between compatibility and task version (estimate ± se = 0.608 ± 0.314, *χ2* = 3.74, *df* = 1, *p* = 0.053) nor the main effect of task version (estimate ± se = − 0.492 ± 0.239, *χ2* = 1.21, *df* = 1, *p* = 0.271) were significant. Finally, there was a significant effect of trial number (estimate ± se = − 0.492 ± 0.239, *χ2* = 9.217, *df* = 1, *p* = 0.002), meaning that subjects improved their performance through the session.

**Table 2:** Experiment 2: GLMM of proportion of correct trials in the Half task predicted by compatibility.

	Estimate	SE	CI lower	CI upper	Model stability min	Model stability max	*χ2*	*df*	*p*^1,2^
Intercept	1.310	0.262	0.825	1.898	1.310	1.149			
Task version (correct no-responses rewarded)	− 0.492	0.239	− 1.021	− 0.051	− 0.492	− 0.682	1.209	1	0.271
Compatibility (incompatible)	− 1.116	0.301	− 1.797	− 0.551	− 1.116	− 1.282	7.185	1	**0.007**
Trial number^4^	0.237	0.079	0.091	0.398	0.237	0.199	9.217	1	**0.002**
Task order^4^	− 0.004	0.083	− 0.157	0.158	− 0.004	− 0.067	0.002	1	0.965
Task version (correct no-responses rewarded)*compatibility (incompatible)	0.608	0.314	− 0.001	1.250	0.608	0.410	3.742	1	0.053

^1^Statistically significant p-values are shown in bold.

^2^Some p-values are not indicated due to their limited interpretation.

^3^Reference categories: task version = correct no-responses not rewarded; compatibility = compatible.

^4^Predictors were z-transformed to a mean zero and a standard deviation of one. Original mean(sd): trial number = 20.500 (11.551); task order = 1.5 (0.500).

## Discussion

While brown capuchin monkeys showed a compatibility effect in a computer-based Joint Simon task, as they did in a manual version of the task^18^, their behavior in the updated control conditions (Half and Social facilitation tasks) challenges the interpretation of this as a social phenomenon. In Experiment 1, as has been found previously, the compatibility effect appeared only when subjects played together in the same computer, but not when their partner was not present, or present but not engaged in the task, which has been traditionally interpreted as evidence of action co-representation. Nevertheless, after we ensured that subjects understood the control task by training them on a Go-No Go (GNG) paradigm (Experiment 2), we observed the same compatibility effect in the control Half task, arguing against a social account of the compatibility effect in the Joint Simon task. Importantly, previous research with non-human primates shares the same limitation as our Experiment 1, suggesting the need for re-examination of previous claims of action co-representation in non-human primates. We discuss each of these findings in more detail below.

In Experiment 1 we found that the presence of a compatibility effect depended on the condition. That is, monkeys succeeded more in compatible (stimulus target and correct response are in the same side) vs incompatible trials (stimulus target and correct responses are in opposite sides), but this effect was present only in the solo Simon task, in which they were responsible for the whole task, and in the Joint Simon task, in which one individual was responsible for half of the task while their partner was responsible for the other half. We did not find a compatibility effect in the conditions in which subjects performed only half of the task without a partner (Half task), or with a partner present but not engaged in the task (Social facilitation task). This pattern has been argued to be evidence of action co-representation of their own and their partner’s part of the task^12^. However, this claim must be supported by showing that the compatibility effect disappears when the partner is removed. Consequently, the Half task is crucial in interpreting the results, and most of the debate about whether the compatibility effect in the Joint Simon task is a social phenomenon revolves around this task^32^. However, in order for the control (Half task) to be valid, it is necessary that the subject understands the contingencies of the task. Based on the inspection of the data of Experiment 1, we realized that this might not be the case, raising doubt about its validity as a control condition.

We found in our data that the capuchin monkeys selected a response in virtually every trial in the Half (and Social facilitation) task. This was problematic because, in order to be a compelling control condition for the Joint Simon task, subjects would have to understand that the Half and Social facilitation tasks were meant to be played as GNG tasks. Given our results, this did not seem to be the case. We considered two possibilities to explain this failure to refrain from selecting the incorrect answer in Experiment 2. First, because subjects could not obtain a reward on the trials in which the correct box was not available, subjects could have persisted in selecting a response to promptly finish the trial and initiate a new one. This behavior could have shortened the time interval to receiving a food reward. While we considered this unlikely, because selecting the incorrect response led to a time-out, we were able to definitively refute this explanation because in Experiment 2, subjects gave a similar number of no-responses in the two versions of the Half task, independent of whether they could receive a reward in the trials in which the correct response box was not available.

Our results point to the second explanation: subjects simply did not understand that the task is a Go-No Go (GNG) task. Indeed, when subjects were trained in a GNG before the Half task, in Experiment 2, they then performed as humans typically do in this task, that is, selecting a response only when the correct response was available^33^. This made the Half task in Experiment 2 an appropriate control condition for the Joint Simon task. Critically, after training, capuchin monkeys showed compatibility effect in the Half task and social facilitation task. Since this cannot possibly be due to action co-representation, as there is no partner engaged in the same task, some other element or elements of the setup, rather than the partner’s engagement in the joint task, must underlie the effect in Experiment 2 and, we presume, in Experiment 1. We cannot conclusively identify what this element is based on the current data but believe that our results can be explained by the fact that the monkeys respond using a joystick.

Traditionally, human participants are tested using buttons or keyboards to respond and must choose whether to press the left or the right key in the full Simon task, creating the conflict between response and stimulus. Conversely, in the Half task the spatial dimension of the response is less salient because there is only one key that they choose to push or not, thus there is no spatial choice. However, in our case, even in the Half task, subjects had to push the joystick to one specific direction to select a response, which may lead them to code the response in spatial terms. This, in turn, could have created a conflict between the spatial position of the response and the stimuli target in incompatible trials. Indeed, other studies in humans have also found a compatibility effect in GNG tasks^34,35^. Specifically, Dittrich et al^34^ showed that when the spatial dimension of the response was made more salient, which they also did by using a joystick, there was a compatibility effect in the GNG (Half task).

Alternatively, it is possible that the compatibility effect that we found in the Half task in Experiment 2 was a carry-over effect from Experiment 1, in which they might have formed a strong association between the stimulus and the spatial responses that could not inhibit in the Half task^36,37^. Nevertheless, we deem this explanation unlikely, because in Experiment 2 all subjects showed that they overcame the association between stimulus-spatial responses by successfully completing the GNG training.

Considering all the above, our findings are inconsistent with a co-representation account of the compatibility effect. This is in line with the long lasting debate about the social nature of the Joint Simon^6,24,32,38^. Thus, the challenge is to find a theoretical framework that can shed light on why the compatibility effect appears only in certain circumstances. One of these approaches is the spatial response coding account^21,23^, which proposes that the spatial components of the task affect how the participants encode their response spatially, ultimately causing the compatibility effect. This can explain the compatibility effect in our study because the use of a joystick made the spatial dimension of the response more salient in all the conditions.

While we have focused our discussion on the limitations and interpretation of our study’s Half task in particular, these conclusions also extend to other non-human primates. So far, all the studies using the Joint Simon task in non-human primates have shown the same problem as our Half task: subjects do not seem to understand the task as a GNG, and respond to virtually every trial^17,18^. Perhaps if subjects in these studies had understood when they were to refrain from answering, the results would no longer have supported the co-representation account, as in our study. Clearly there is a compelling need for control tasks that effectively control for alternative explanations before we can interpret the compatibility effect in the Joint Simon task as evidence of action co-representation in non-human primates.

Unfortunately, we could not answer the question of whether capuchin monkeys co-represent the role of their partner in computer-based settings; although we have no evidence against that idea, our findings question the validity of the Joint Simon task as a measure of co-representation. There are, however, different methodologies that might help determine how non-human primates understand –or not– the social nature of computer-based joint tasks. For example, when playing a computer-based Prisoner's Dilemma, rhesus macaques tended to cooperate more and to adjust their choices to their partner’s previous choice (i.e. cooperative choices were less likely after a partner’s defection and more likely following mutual cooperation) when paired with a conspecific, but not when playing against a computer algorithm^39^. Moreover, when pairs of subjects make choices in these games they show activation of brain areas related to social decision-making^40,41^. Such areas are different from the ones activated when the decisions are based on previous outcomes rather than social information^39^. The distinction observed between the macaques’ behavior and neural activation in social and non-social computer games demonstrates that their performance is not only motivated by reward maximization. However further investigation is needed to address whether this is true for other non-human primate species, and whether these differences can be attributed to action co-representation or whether lower level processes can also explain these results. While challenging, this venture has the potential to deepen our understanding of the evolution and mechanisms underlying cooperation across the animal kingdom.

## Methods

### Subjects

We tested 10 adult capuchins (n = 7 dyads; age: *M (SD* ) = 18(5.66), range: 9–25 years) socially housed in multi-male, multi-female groups made up predominantly of female matrilines at the Language Research Center of Georgia State University. Subjects included two females and one male (paired in two dyads) from one group, three females and one male (paired in three dyads) from a second group, and three females (paired in two dyads) from another group. All subjects were previously trained to voluntarily enter individual testing chambers and participate in computer-based cognitive tasks using a joystick to control a cursor on the screen^42^. Some of these tasks involved a joint computer screen with two players^29,43^. They could choose not to enter the testing boxes without any consequences other than missing out on the potential value of participating in the test. Throughout the experiment, monkeys were automatically rewarded for correct choices with 45 mg banana-flavored pellets (Bio-Serv, Frenchtown, NJ, U.S.A.). Testing sessions typically ranged from 20 min to two hours, the length of which was determined by how quickly subjects completed the trials. All monkeys had ad-libitum access to water at all times, including during test sessions, and they were never deprived of food, water, or outdoor and social access to encourage testing.

### General procedure

We designed a Simon task in which one stimulus (an orange circle or purple triangle) was shown on a computer screen, either on the right or the left side of the screen. Subjects were required to ignore the stimulus location and to respond only to the stimulus type by moving the cursor toward one of two response boxes. The correct response was always to move the cursor to the right box if a purple triangle was shown, and to move the cursor to the left box if an orange circle was shown. All trials began with the start screen, which displayed a gray start button that subjects had to select (moving the cursor inside the box). Once the start button was selected, the response boxes (or box, see details for each condition below) appeared, together with the stimulus target (purple triangle or orange circle). Once the capuchins selected a response or after 5 s, whichever came first, the screen went white and all the icons (stimulus, response boxes, and cursor) disappeared from the screen. If the correct response box was selected, subjects received positive auditory feedback and one banana-flavored pellet. In the case of incorrect responses, subjects received negative auditory feedback followed by a 5-s timeout. With some exceptions (see below), no-responses were treated as incorrect choices. All trials were followed by an inter-trial interval of one second, after which the start screen reappeared, and a new trial could be started. Through all the training and test phases, the stimulus target was shown in pseudorandomized order, ensuring that no stimulus was shown more than three consecutive times and that each stimulus (or stimulus-position combination in the test conditions) appeared an equal number of times in each block.

#### Experiment 1: training phase

During training, subjects had to move the cursor towards one of two response boxes depending on which stimulus was shown in the center of the screen. The first step of training consisted of 20 forced-choice trials: 10 trials in which the stimulus target was the purple triangle, followed by 10 trials in which the stimulus target was the orange circle. In the forced-choice trials the cursor on screen could only move in the direction of the correct response box, preventing subjects from making mistakes. In the second step of training, capuchins completed blocks of 40 trials in which they could move the cursor to any of the two response boxes. Subjects were allowed to complete as many training blocks as they wanted within a testing day. Training was finished once they reached at least 80% accuracy in two consecutive blocks.

#### Experiment 1: testing

Each testing session started with a short pre-test phase, aimed to ensure that subjects still remembered the correct response to each stimulus. The pre-test phase was a shortened version of the training task, in which subjects first completed 20 forced trials (10 for each stimulus), followed by blocks of 10 trials identical to the second phase of training. Once they responded correctly 8 out 10 trials in one block, they advanced to testing. Testing consisted of 40 test trials. In contrast to the training (in which the stimulus appeared at the center of the screen), in the test trials the target stimulus could be either shown in the left or the right side of the screen. Thus, there were two types of trials, depending on whether the target stimulus was shown on the same (compatible trials) or opposite (incompatible trials) side of the screen as the correct response box. Monkeys participated in four different conditions:

- *Simon task*: Subjects were individually tested and were required to move the cursor to the right or the left response box depending on the stimulus shown.
- *Half task:* Subjects were individually tested and only one of the two response boxes was available on screen (right or left box, counterbalanced between individuals). To keep this condition similar to the manual version of the task^17,18^, in which subjects did not have access to one of the pulling drawers, we made the response box invisible and we limited the mobility of the cursor so that subject could not move the cursor towards the area in which the unavailable response box was supposed to be. That is, subjects could freely move the cursor on one half of the screen (in which the available response button was) but, if the cursor passed one centimeter into the other half, their cursor automatically bounced back to the center. (see Fig. 2). For example, if a given subject has the right response box available, and in their case this box corresponded to the purple stimulus, then they have to select it when the purple stimulus is shown (“go trial”), and should not react when the orange stimulus is shown (“no-go trial”). In the go trials, subjects received feedback for their response (correct if they selected the response box, incorrect if they did not select a response), as described in the general procedure. However, in the no-go trials, they received negative feedback if they selected the available box (negative auditory feedback followed by a 5-s time out) but did not receive any feedback if they refrained from selecting any response (i.e. the next trial started after the inter-trial interval, without any food reward, acoustic feedback, or time out).
- *Joint Simon task*: Subjects were tested in pairs. From the subject’s point of view, the task was identical to the Half task, with the exception that two individuals played on the same computer, with one screen equidistant from both, so each individual was responsible for one complementary half of the task (either pressing for the purple or orange stimulus). Additionally, to enhance the joint nature of the task, both individuals were rewarded for each correct response.
- *Social facilitation task*: Subjects were tested individually and the task was the same as the Half task, except that the computer was positioned in the same place as in the Joint Simon task (between subject and partner) and the partner was participating in a (different) computer task on a separate computer. This task aimed to control for the possible effect of the presence of a partner who was also using a joystick.

Subjects experienced each condition in each round of testing, and two rounds of testing (i.e., ultimately experiencing two sessions of each condition). Rounds were consecutive, that is, after they completed one testing session of each condition, they were tested again in all conditions. The order of the conditions within each round was counterbalanced and randomized. Each condition consisted of 40 trials that subjects typically finished within one testing session. If subjects were unable to finish the 40 trials within one testing session, we then repeated the same test again the next day to finish the remaining trials, until we had at least 40 total trials across two consecutive testing days. This happened in the two Joint Simon tests with one dyad (Nkima and Gambit). If subjects were still unable to complete 40 trials within two consecutive testing days after three attempts, they were removed from that condition. This happened with one female (Nala) that stopped participation after the first test in the second testing round.

#### Experiment 2: training phase

In Experiment 2 subjects underwent a series of training phases to learn how to perform a Go/No-go task. For this, they had to select a response box or to refrain from selecting that box depending on the type of stimulus shown in the center of the screen. Both the stimuli and the response box (right or left) that were available for each individual were the same as in the Half task in Experiment 1. Subjects could also freely move the cursor on one half of the screen, but again it would automatically go back to the center when they tried to move it to the other half of the screen. However, unlike Experiment 1, here subjects were rewarded for both selecting the response box when the corresponding stimuli was shown and for not selecting a response when the correct response box was not available.

The first step of training consisted of 20 forced trials: 10 trials in which the stimulus target was the purple triangle, followed by 10 trials in which the stimulus target was the orange circle. In these forced trials, the cursor mobility in the screen was limited, preventing the subjects from making any mistakes. That is, in the trials in which the correct response box was not available, the cursor would stay static in the center of the screen. In the trials in which the correct response box was available, the cursor could only move towards that box (i.e., cursor would not respond if the subjects tried to move it away from the correct response). In the second step of training, capuchins completed blocks of 40 trials in which they could move the cursor freely.

Because inhibiting a response can be challenging for non-human primates, all subjects underwent stepwise training to slowly build them up to not select any target (when the correct response box was not available) for 5 s, which was the final duration used in testing. First, subjects were only given 1 s to respond (or not respond). Once subjects completed two consecutive blocks with at least 80% of accuracy with this duration, the program automatically started a new block increasing this latency to 2 s. This process repeated until the subjects were successfully not responding for up to 5 s, at which point they passed criterion.

#### Experiment 2: testing

As in Experiment 1, each testing session started with a pre-test phase consisting of 20 forced trials (10 for each stimulus), followed by blocks of 10 trials identical to the last phase of training. The pre-test phase was completed once subjects succeeded in 8 out of 10 trials in one of these blocks. After the pre-test phase, the 40 test trials started. In the test trials the target stimulus was shown either on the left or the right side of the screen, resulting in compatible and incompatible trials. Monkeys participated in two different versions of the task:

- *Half task with unrewarded correct no-responses:* This task was identical to the Half task in Experiment 1.
- *Half task with rewarded correct no-responses:* This task was nearly identical to the Half task in Experiment 1 except subjects were also rewarded for correct no-responses, as in the training.

### Statistical analysis

All models were fitted in R version 4.2.3^44^, using the function *lmer* of the R-package *lme4*^45^.

In Experiment 1 we explored whether there was a compatibility effect depending on the condition. To test this, we ran a Generalized Linear Mixed effect Model (GLMM) with accuracy as the response variable (whether subject’s choice was correct or incorrect in each trial, binomial error distribution) and the interaction between compatibility (whether the trial was compatible or incompatible) and condition as predictor. We also included the individual effects of compatibility and condition, and the fixed effects of trial number and session number (whether it was the first or second session of each test).

In Experiment 2, we tested whether there was a compatibility effect in the Half task after the capuchins were trained in a Go/No-go task. For this, we ran a GLMM with accuracy as the response variable and trial’s compatibility as the predictor. We also included the fixed effects of task version (with or without rewarding the correct no-responses), the interaction between task version and compatibility, the trial number, and the task version order (first or second).

To keep Type I error rates at the 5% level and account for pseudo-replication^46^, we included the random intercept of Subject ID and the theoretical identifiable random slopes (in Experiment 1: condition, compatibility, trial number, and session; in Experiment 2: task version, compatibility, trial number, and task version order) together with the correlations between the random intercept and the random slopes in both of our models. However, we excluded this correlation in both models to reduce model complexity, which resulted in a small reduction in model fit both in Experiment 1 (model with correlations: *logLik* = − 1869.37, *df* = 38; model without correlations: *logLik* = − 1871.704, *df* = 17), and Experiment 2 (model with correlations: *logLik* = − 485.958, *df* = 21; model without correlations: *logLik* = − 490.402, *df* = 11). In Experiment 1, we further excluded the random slopes of trial number and session because it was estimated to be zero, which is indicative of them to be unidentifiable^47^, with no impact on the model fit (model without random slopes of trial number and session: *logLik* = − 1871.704, *df* = 15). For the same reason, in Experiment 2 we also removed the random slopes of trial number and task version order, with no impact in the model fit (model without random slopes of trial number and task version order: *logLik* = − 490.402, *df* = 9).

All numeric fixed effects (trial number, session, order) were z-transformed to a mean of zero and a standard deviation of one to ease convergence and interpretability. Condition and task version were manually dummy-coded before its inclusion in the random slopes.

To prevent inflating Type I error due to multiple testing we used a full-null model comparison approach^48^, testing the significance of the full models compared with models lacking the predictors (interaction between compatibility and condition in Experiment 1; and compatibility in Experiment 2) but otherwise identical to their respective full models. We took a more conservative approach and excluded only the predictors in the null model instead of all the fixed effects because our models were question-driven. Thus, we were interested in a full-null model comparison that could tell with one unique p-value whether our predictor (instead of *any* of the fixed effects) had an effect on the response variable. We did this comparison by means of likelihood ratio test^49^ using the function *anova* with the argument “test” set to “Chisq”. P-values for each term in the model were obtained comparing the full model with a reduced model excluding each particular term, with the function *mixed* of the R-packages *afex*^50^. When a factor or interaction of factors was significant, we computed post-hoc comparisons of contrasts with multivariate t adjustment, using the function *emmeans* or the R-package emmeans^51^.

We assessed the models’ stability with a function kindly provided by Roger Mundry, by comparing the estimates obtained in our full models with those obtained from models with the levels of random effects excluded one at a time. This revealed the models to be fairly stable. To rule out collinearity we inspected the Variance Inflation Factors (VIF) with the function *vif* of the R package *car*^52^ applied to a standard linear model excluding interactions and random effects. This analysis showed no problems of collinearity between the variables (all VIF were approximately 1). We calculated the confidence intervals using parametric bootstrap, using the function *confint.merMod* of the R-package *lme4*. Finally, we draw the plots using the R-package *ggplot2*^53^ (Supplementary Information S1).

### Ethics statement

All protocols involved in this study were approved by the Georgia State University IACUC (#A20018 and #A23018). Additionally, all protocols and procedures used in this study were in accordance with the relevant legal requirements governing animal research in the United States of America and the American Psychological Association ethical standards for the treatment of animals in research and the American Society of Primatologists statement on the Principles for the Ethical Treatment of Non-human Primates. GSU is fully accredited by AAALAC. The project reporting complied with ARRIVE guidelines.

### Supplementary Information

Supplementary Information.

## Data Availability

All data generated or analyzed during this study are included in the Supplementary Information files.
